# A review on hypo-cholesterolemic activity of *Nigella sativa* seeds and its extracts

**DOI:** 10.6026/97320630018343

**Published:** 2022-04-30

**Authors:** Karmegam Uma Maheswari, K Dilara, S Vadivel, Priscilla Johnson, Selvaraj Jayaraman

**Affiliations:** 1 Department of Physiology, Sri Ramachandra Medical College and Research Institute (SRMC & RI), Porur, Chennai- 600116, India; 2 Department of Physiology, Karpaga Vinayaga Institute of Medical Education and Research, Madhuranthagam, Chengalpet-603308, Tamil Nadu, India; 3 Department of Biochemistry, Saveetha Dental College & Hospital, Saveetha Institute of Medical & Technical Sciences, Chennai 600077, India

**Keywords:** *Nigella sativa*, black seed, hypo cholesterolemic activity

## Abstract

*Nigella sativa* (N. sativa) (Family Ranunculaceae) is a popular therapeutic herb in many parts of the world. It is widely used in traditional medical systems such as Unani, Ayurveda and Siddha. Seeds and oil have a long history of folkloric use in many medicinal and culinary systems. The seeds of N. sativa have long been used to treat a variety of illnesses and disorders. Studies on N. sativa and its therapeutic potential have been investigated. This includes anti-diabetic, anticancer, immune-modulatory, analgesic, antimicrobial, anti-inflammatory, spasmolytic, bronchodilator, hepato-protective, renal protective, gastro-protective, antioxidant properties, and several others. *Nigella sativa* contains thymoquinone. This is a bioactive component of the essential oil with medicinal benefits. Therefore, it is of interest to report a comprehensive data on the therapeutic usefulness of N. sativa in hypo-cholesterolemic activity.

## Background:

Cholesterol is a necessary component of all living organisms; without it, the organism couldn't work efficiently and would die [1]. One of the most important cardiac risk factors has been identified as elevated plasma cholesterol (hypercholesterolemia). Serum cholesterol levels are directly associated to cardiac morbidity and mortality. Many studies have found that high serum cholesterol levels alter the biochemical properties of blood components and artery intima, facilitating the progression of atherosclerosis. [2]. Hypercholesterolemia has been shown to produce oxidative stress by inducing free radical-mediated lipoprotein peroxidation. This stress is caused by a mismatch between the production of free radicals and the antioxidant defense system's efficacy. The activity of antioxidant enzymes have been found to be abnormal in those who have cardiovascular disease [3]. Lowering cholesterol levels was the prime goal in preventing the occurrence of coronary heart disease (CHD). Cholesterol lowering and dietary modifications have also been included in health care strategies to guard against CHD [4]. The optimal intake to keep preferable blood lipids levels, which could protect against heart disease, has attracted people's curiosity. Elevated levels of serum total cholesterol, low density lipoprotein cholesterol (LDL), and triglyceride have indeed been associated with a greater risk of heart disease, whereas elevated levels of high-density lipoprotein cholesterol (HDL) have indeed been related to a lower risk of heart disease [5]. As a result, a dietary pattern that successfully lowers TC, LDL, and triglyceride levels while preserving or increasing HDL is preferred.

Aside from medicine, diet composition plays an essential role in blood lipid and lipoprotein concentration regulation. Plant-based medicines are well-known for their therapeutic benefits because they have little or no adverse effects [6]. Traditional remedies have gained a lot of popularity in various parts of the world during the last 20 years or so. Extensive study on different plant species' medicinal principles and potential is causing traditional medicines all around the world to be reevaluated. Despite advances in conventional chemistry and pharmacology in the development of successful pharmaceuticals, the plant kingdom may still be a useful source of new medicines. Isoflavones, phytosterols, saponins, fibers, polyphenols, flavonoids, and ascorbic acid are only a few of the metabolites produced by plants, and their role in lipid and antioxidant metabolism has sparked attention [7]. Since several studies demonstrated its wide spectrum of pharmacological potential, Nigella sativa (N. sativa) (Family Ranunculaceae) is emerging as a wonder herb with a rich historical and religious heritage. Black seed is the popular name for N. sativa. *Nigella sativa* is a plant native to Southern Europe, North Africa, and Southwest Asia that is grown in a variety of nations across the world, including the Middle East Mediterranean region, South Europe, India, Pakistan, Syria, Turkey, and Saudi Arabia [8]. *Nigella sativa* seeds and oil have been utilized for millennia in the treatment of a variety of diseases all throughout the world. It is also a significant medicine in Indian traditional medical systems such as Unani and Ayurveda [9]. Crude oil extracted from the seeds of N. sativa demonstrated a wide range of therapeutic actions. *Nigella sativa* seed oil can also help with headaches, flatulence, blood homeostasis issues, rheumatism, and other inflammatory disorders [10]. Therefore, it is of interest to report a comprehensive data on the therapeutic usefulness of N. sativa in hypo-cholesterolemic activity.

## Chemical composition of black seeds:

Since then, the several active chemicals have been extracted, recognized, and published in various black seed types. Thymoquinone (30% -48%), thymohydroquinone, dithymoquinone, p-cymene (7% -15%), carvacrol (6% -12%), 4-terpineol (2% -7%), t-anethol (1% -4%), sesquiterpene longifolene (1% -8%), -pinene, and thymol are the most important active chemicals. In trace proportions, black seeds also contain additional chemicals. Isoquinoline alkaloids, such as nigellicimine and nigellicimine-N-oxide, and pyrazol alkaloids, or indazole ring containing alkaloids, such as nigellidine and nigellicine, are found in seeds. Furthermore, the seeds of N. sativa contain alpha-hederin, a water-soluble pentacyclic triterpene, as well as saponin, a possible anticancer agent [11].

## Atherosclerosis activity:

The most common cause of sickness and mortality in the world is atherosclerosis. [12 - Check with author] It is a complex disease with numerous risk factors. Atherosclerosis is caused by a combination of causes, one of which is a high level of cholesterol. Lowering cholesterol levels with medication or dietary changes may lower the risk of coronary heart disease (CHD) [13]. Several plants have been identified in traditional medicine, with some of them delivering excellent relief to those suffering from cardiovascular illnesses. Botanical dietary supplements can help your heart health and prevent atherosclerosis in several ways [13]. Many herbal substances have been found in recent research to lower plasma triacylglycerol (TG) and total cholesterol (TC) levels while increasing high-density lipoproteins (HDL) levels, lowering the risk of coronary heart disease (CHD)[12 - check with author]. *Nigella sativa* (NS) has been found to provide a variety of health benefits, including hypoglycemic, hypocholesterolemic, and antioxidant properties [14]. The choleretic impact of NS is a key mechanism that may explain the plant's lipid-lowering and atheroprotective benefits. Treatment with NS seed powder and seed oil was found to lower blood total and LDL cholesterol levels while increasing HDL cholesterol levels in hypercholesterolemic rabbits in one research. NS's lipid-modulating effects were accompanied by smaller atherosclerotic plaques and a lower intima/media ratio [15].

The impact of N. sativa seeds powder (1000 mg/kg) and oil (500 mg/kg) on atherosclerosis in diet-induced hypercholesterolemia rabbits was studied for eight weeks in comparison to simvastatin (10 mg/kg). The results group treated with N. sativa seeds powder or oil considerably decreased arterial wall lipid deposition, TC and LDL, and increased HDL. Furthermore, plaque development halted and lowered the intima/media ratio significantly [16 - check with author]. In diet-induced hypercholesterolemia rabbits, N. sativa seeds powder (100 mg/kg/day) dramatically decreased serum levels of TC, TG, and LDL-C and elevated HDL-C during a four-week period [17]. Arachidonic acid (AA)-triggered platelet accumulation and blood coagulation were inhibited by the methanol soluble part of the N. sativa seed oil. 2-(2-methoxypropyl)-5-methyl-1,4-benzenediol, thymol, and carvacrol, among other separated oil constituents, had a substantially higher effect on AA-triggered platelet accumulation and blood coagulation than aspirin [18]. Furthermore, fatty streak production in the left and right coronary arteries, as well as the aorta, was dramatically reduced. According to the findings, N. sativa's anti-inflammatory and antioxidant capabilities may be responsible for this impact [19]. In hypercholesterolemia rabbits, therapies with honeys (a resinous hive product obtained by honeybees from diverse plant origin) and TQ resulted in significant reductions in serum TC, LDL-C, triglycerides, and thiobarbituric acid-reactive concentrations, as well as increased HDL-C and glutathione content. Propolis and TQ had a preventive role against hypercholesterolemia-induced aortic tissue damage, according to histopathological analysis. The findings also revealed that antioxidant mechanisms may be involved in the protective effects of propolis and TQ [20]. Table 1(see PDF) illustrates the antiatherogenic and anti-platelet actions of N. sativa and its components.

## Effect *Nigella sativa* seed extracts in animal studies:

The effects of methanol extracts (810 mg/kg) and volatile oil (410 mg/kg) of N. sativa seed on hyperlipidaemia rats revealed that therapies with plant considerably decreased plasma triglycerides (TG), total cholesterol (TC), extremely low density lipoproteins (VLDL-C), low density lipoproteins (LDL-C), -hydroxy-methylglutaryl-CoA reductase activity, and improved higher densities lipoproteins (HDL-C) concentration [21]. Intra-gastric gavage of N. sativa seed petroleum ether extract decreased fasting plasma insulin and TG levels while increasing HDL-C [22]. Besides that, oral feeding of N. sativa seed fixed oil (1 ml/kg) to rats for 12 weeks decreased TC, TG, hyperglycemia, leukocytes, and platelet counts while increasing hematocrit and haemoglobin levels [23]. In rats, therapies with N. sativa seed oil (800 mg/ kg, p.o. for 4 weeks) reduced blood TC, LDL, and TG while increasing serum HDL. Medication with N. sativa (50, 100, 200, 300, 400, 500 mg/day) reduced blood TC, LDL, and TG while enhancing the HDL/LDL ratio in normal rats. [24]. An ethanolic extract of N. sativa seed (0.5, 1 and 1.5 mg/kg, ip) was shown to prevent adrenaline-induced dyslipidemia and left ventricular hypertrophy in rats. In adrenaline-induced dyslipidaemia mice, injection of the plant for two weeks significantly reduced TC, TG, LDL-C, and elevated HDL-C. Furthermore, eight weeks of treatment with N. sativa boosted antioxidative activity and reduced left ventricular hypertrophy and cardiomyocyte size. [25].

N. sativa In Sprague Dawley rats, (1000 mg/kg/day) exhibited a significant reduction in TC, TG, LDL-C, and an elevation in HDL-C when compared to simvastatin, a synthetic antihyperlipidemic medication. The findings indicated that N. sativa has the potential to be employed as an antihyperlipidemic medication with no negative side effects. [26]. The impact of N. sativa seed squashed therapy (7.5 g/kg/day) on TC, TG, LDL-C, and MDA was been observed in a rabbit model of hyperlipidemi. Furthermore, treatment with N. sativa (5%) significantly reduced TC and LDL-C in hypercholesterolemia rabbits. [27]. The impact of several N. sativa extracts on lipid levels in ovariectomized as an experimental animals of menopause has been examined, and the findings show that distinct N. sativa extracts considerably lowered sugar levels and LDL-C, however variations in TC, TG, and HDL-C were not significant [28]. Data shows that treatment with either N. sativa (0.4 mg/kg) or olive oil (0.4 mg/kg) drastically diminished TC, TG, LDL-C, and VLDL-C in a mouse model of hyperlipidemia. The value of HDL-C in the olive oil group, on the other hand, was considerably higher than in the N. sativa group. [29]. The effects of N. sativa acetone extraction (0, 0.2, 0.4 percent) and N. sativa seed powder (0, 1.5, 2.5, 3.0 percent) on hyperlipidaemia laying hens over 4 weeks demonstrated that feeding either with the seed powder or acetone extracts of the plant seeds significantly lowered TC and TG. [30]. In Pekin ducklings, supplementing with N. sativa seed (2%) decreased HDL-C while increasing TC, TG, LDL-C, and VLDL-C. [31]. Canola oil and N. sativa seed powder considerably lowered TC and LDL-C while increasing HDL-C in a non-significant way [32]. In addition, therapy with N. sativa (30 mg/kg, po) enhanced HDL-C while decreasing LDL-C [33]. In albino rats, palm oil enhanced TC and LDL-C levels and reduced HDL-C levels at 24 weeks, and though therapies with N. sativa dramatically decreased TC and LDL-C levels and increased HDL-C levels [34]. In hyper cholesterolemia rabbits, feed with 5% N. sativa dramatically reduced arterial wall lipid accumulation, TC, and LDL. TQ (10 mg/kg) lowered TC, TG, and LDL-C while increasing HDL-C in a rabbit model of atherosclerosis for eight weeks. The alterations in lipid profile were not significant. The impacts of N. sativa and also its constituents on lipid profile were seen in Table 2(see PDF).

## Role of N. sativa in clinical studies:

The impacts of N. sativa seed powder on blood cholesterol, HDL, LDL-c, and TG in menopausal women of 2 categories were explored in a clinical research. The treatment group got N. sativa powder (500 mg) capsules, while the placebo group got placebo capsules (wheat germ, 100 mg). For a period of two months, capsules of N. sativa powder have been given orally at a quantity of 1 g after breakfast daily. According to the data, N. sativa significantly increased serum HDL-C while significantly lowering LDL-C, TC, TG, and FBG [35]. In people with metabolic syndrome, treatment with N. sativa seed oil considerably elevated blood HDL-C and lowered LDL-C [36]. In hypercholesterolemia patients, oral dose with N. sativa powder at a dose of 1 g everyday prior to actually breakfast for 2 months lowered blood levels of TC, LDL, and TG while increasing HDL [37]. In addition, after months of therapy with N. sativa powder (500 mg) and statin (10-20 mg) in people with acute coronary syndrome in Multan, Pakistan, serum TC, LDL, and TG levels were considerably lower than in the statin (10-20 mg) alone group [38]. The effects of an eight-week oral administration of N. sativa seed in male people with moderate dyslipidemia and high blood pressure were also studied. Patients were randomly categorized into three groups: a placebo, 100 mg of N. sativa extract two times a day, or 200 mg of N. sativa extract two times a day. The serum levels of TC, TG, LDL, SBP, and DBP in N. sativa extract groups decreased significantly in a dose-dependent manner. [39].

The impacts of N. sativa on glucose, uric acid, TG, cholesterol, blood urea nitrogen (BUN), and creatinine in ordinary healthy individuals were studied in two groups: I) the test group received N. sativa powder (500 mg) capsules twice daily, and II) the placebo group got brown sugar (500 mg) capsules twice daily. The findings showed that N. sativa considerably lowered sugar levels and cholesterol levels [40]. Another human investigation found favorable impacts of the capsulated N. sativa powder (500 mg) on blood pressure, serum total cholesterol, LDL cholesterol, triglycerides, and fasting blood sugar, but the results were not statistically significant due to the small sample size. [41]. The impact of N. sativa intake (2 g/day) and aerobic exercises on lipid profile in inactive obese females over an eight-week period also revealed that the plant and aerobics together resulted in huge upgrades in blood LDL-C and HDL-C [42]. The impact of 2.5 ml N. sativa seed oil orally twice in patients with metabolic syndrome was compared to atorvastatin 10 mg once per day, metformin 500 mg twice a day, atenolol 50 mg once per day, and amlodipine 5 mg daily for at least a period of six weeks. Treatment with N. sativa greatly reduced blood levels of LDL-C and improved HDL-C [43]. In healthy patients, taking 2.5 ml N. sativa seed oil two times a day for 8 weeks lowered fasting blood cholesterol, LDL, TG, glucose, and HbA1C levels considerably [44]. Furthermore, obese women who took 3 g of N. sativa seed oil every day for two months saw a substantial reduction in TG, VLDL, weight, and waist circumference [45]. In hyperlipidemia patients, diet with N. sativa 2 g/day for 4 weeks resulted in favorable reductions in blood levels of TC, TG, and LDL [46]. In individuals with hypertriglyceridemia, saboose-asapghol (Plantago ovata) 4 g and N. sativa 2 g two times a day for 90 days dramatically lowered serum TG levels [47]. In hyperlipidemia patients, two tea spoons (about 9.0 g) of N. sativa seed per day compared to gemfibrozil 600 mg twice daily for eight weeks dramatically lowered blood levels of TC, TG, and LDL-C and elevated HDL-C [48].

In hyperlipidemia patients, the benefits of N. sativa were compared to nicotinic acid together with a low-fat diet and physical activity in three groups: For two months, the effects of I) a placebo, II) 2 tea spoons N. sativa after breakfast, and III) niacin 2 g in divided dosages after breakfast, lunch, and dinner were studied. In hyperlipidemia patients, N. sativa and niacin dramatically lowered serum LDL-C levels while increasing HDL-C levels [49]. In hypercholesterolemia, a combination of N. sativa seeds (50 mg/kg) and honey reduced serum TC, TG, TC: HDL-C, as well as SBP, DBP, and elevated HDL-C. In dyslipidemia patients, a combination of N. sativa seeds (500 mg), garlic oil (250 mg), and simvastatin (10 mg) capsules given once daily after dinner lowered serum TC, TG, LDL-C, and Non-HDL while increasing HDL-C [50].

## Conclusion:

According to several findings, N. sativa and its constituents have hypocholesterolemic properties. However, further research is needed to determine the specific molecular and cellular basis of N. sativa's hypo-cholesterolemic properties and the effects of its constituents. Furthermore, more clinical research into the effects of the plant and its ingredients on hypo-cholesterolemic effects was required.
